# External validation of a novel nomogram for diagnosis of Protein Energy Wasting in adult hemodialysis patients

**DOI:** 10.3389/fnut.2024.1351503

**Published:** 2024-08-13

**Authors:** Danying Yan, Yi Wang, Jing Hu, Renhua Lu, Chaoyang Ye, Nanmei Liu, Dongping Chen, Weiwei Liang, Liang Zheng, Wenrui Liu, Tianying Lan, Naiying Lan, Qing Shao, Shougang Zhuang, Xiaoyan Ma, Na Liu

**Affiliations:** ^1^Department of Nephrology, Shanghai East Hospital, Tongji University School of Medicine, Shanghai, China; ^2^Department of Nephrology, Seventh People’s Hospital of Shanghai University of Traditional Chinese Medicine, Shanghai, China; ^3^Department of Nephrology, Ren Ji Hospital, School of Medicine, Shanghai Jiaotong University, Shanghai, China; ^4^Department of Nephrology, Shuguang Hospital Affiliated to Shanghai University of Traditional Chinese Medicine, Shanghai, China; ^5^International Medicine III (Nephrology & Endocrinology), Naval Medical Center of People's Liberation Army of China, Naval Medical University, Shanghai, China; ^6^Key Laboratory of Arrhythmias of the Ministry of Education of China, Research Center for Translational Medicine, Shanghai East Hospital, Tongji University School of Medicine, Shanghai, China; ^7^Department of Medicine, Rhode Island Hospital and Alpert Medical School, Brown University, Providence, RI, United States

**Keywords:** Protein Energy Wasting, diagnosis, prediction model, external validation, hemodialysis

## Abstract

**Background:**

Protein Energy Wasting (PEW) has high incidence in adult hemodialysis patients and refers to a state of decreased protein and energy substance. It has been demonstrated that PEW highly affects the quality of survival and increases the risk of death. Nevertheless, its diagnostic criteria are complex in clinic. To simplify the diagnosis method of PEW in adult hemodialysis patients, we previously established a novel clinical prediction model that was well-validated internally using bootstrapping. In this multicenter cross-sectional study, we aimed to externally validate this nomogram in a new cohort of adult hemodialysis patients.

**Methods:**

The novel prediction model was built by combining four independent variables with part of the International Society of Renal Nutrition and Metabolism (ISRNM) diagnostic criteria including albumin, total cholesterol, and body mass index (BMI). We evaluated the performance of the new model using discrimination (Concordance Index), calibration plots, and Clinical Impact Curve to assess its predictive utility.

**Results:**

From September 1st, 2022 to August 31st, 2023, 1,158 patients were screened in five medical centers in Shanghai. 622 (53.7%) hemodialysis patients were included for analysis. The PEW predictive model was acceptable discrimination with the area under the curve of 0.777 (95% CI 0.741–0.814). Additionally, the model revealed well-fitted calibration curves. The McNemar test showed the novel model had similar diagnostic efficacy with the gold standard diagnostic method (*p* > 0.05).

**Conclusion:**

Our results from this cross-sectional external validation study further demonstrate that the novel model is a valid tool to identify PEW in adult hemodialysis patients effectively.

## 1 Introduction

As the prevalence of long-term diseases such as diabetes mellitus and hypertension continue to increase accompanied with the aging of the population, the incidence of end-stage renal disease (ESRD) has also risen dramatically and has become a widespread public health problem (1–4). Hemodialysis is the most frequently practiced renal replacement therapy for patients with ESRD (5). Nevertheless, patients with chronic kidney disease (CKD), especially those with ESRD, are susceptible to muscular atrophy, muscle sparing, and cachexia because of the need for long-term maintenance hemodialysis (MHD), leading to anemia, immune dysfunction, poor dialysis tolerance, and frequent infections, and even directly affect the quality of patients’ life and survival rate (6–13).

In 2007, the International Society of Renal Nutrition and Metabolism (ISRNM) officially adopted the term of Protein Energy Wasting (PEW) to describe metabolic and nutritional disorders in chronic disease states (14). PEW refers to a state of declining protein and energy stores in the body, characterized by insufficient dietary nutrient and calorie intake (unintentional low daily protein intake (DPI) < 0.80 g/kg per day for at least 2 months for dialysis patients or DPI < 0.60 g/kg per day for patients with CKD stages 2–5), low body mass index (BMI) (BMI < 23 kg/m^2^, unintentional 5% weight loss over 3 months or 10% weight loss over 6 months, and total body fat percentage < 10%), hypoproteinemia (serum albumin < 38 g/L, serum prealbumin < 0.3 g/L, or serum cholesterol < 1 g/L), micro-inflammatory states, and progressive skeletal muscle atrophy (reduced 5% muscle mass over 3 months or 10% over 6 months, or reduction of mid-arm muscle circumference (MAMC) area over 10% about 50th percentile of reference population) (11, 15, 16). It has previously been documented that 18–75% of the population suffers from varying degrees of malnutrition, especially in patients with ESRD in dialysis, and quality of life and mortality in CKD is closely associated with PEW which has been confirmed by reverse epidemiology (9, 17–20).

In 2008, ISRNM recommended a four-component diagnosis of PEW based on low biochemical markers [serum albumin, prealbumin, or total cholesterol (TC)]; generalized adiposity or weight loss; loss of muscle mass; and insufficient protein or energy intake ratios (14). Unfortunately, in clinical practice, these diagnostic criteria are not easily measured, especially the assessment of protein or energy intake. Even worse, this is probable to hamper the early diagnosis of PEW, to the detriment of improving the prognosis of patients.

In recent years, the study involved in predictive models in the medical field has proliferated. Predictive model is a mathematical formula that determines the risk of a particular outcome based on a person’s predictive variables. The models have gained attention for their potential use in personalized medicine, individualized decision-making, and risk stratification (21). As a result, researchers have developed a large number of tools to predict, score risk, etc. To streamline the diagnostic method of PEW, a novel prediction model was submitted by Chen et al. (22). They identified independent risk factors for PEW through univariate and multivariate logistic regression, and combined them with the diagnostic criteria for ISRNM, resulting in the inclusion of seven influencing factors, BMI, gender, albumin, TC, triglyceride (TG), vitamin D, and N-terminal Pro-B-Type Natriuretic Peptide (NT-proBNP), respectively. Each influencing factor was then valued for its level of value according to the degree of its contribution to the outcome variable (occurrence of PEW). A nomogram was constructed. Ultimately, the predictive value of the incidence of PEW can be calculated from the composite score based on nomogram (22). In addition, they proved that the model had good predictive ability through calibration curves and the Receiver Operating Characteristic (ROC) curve, where the area under the curve was 0.851 (95% CI: 0.799–0.904) (22).

Because predictive models typically perform worse in new patients than in the developed population, models should not be recommended for clinical use until external validity has been established (21, 23). However, we would have to demonstrate that the predictive model also has a high PEW prediction accuracy for different real-life populations, in the sense of externally validating it, before the model can be applied to everyday practice (24–28). The purpose of this study is to conduct an independent external validation of this novel predictive model.

## 2 Materials and methods

### 2.1 Study population

**Figure 1:**
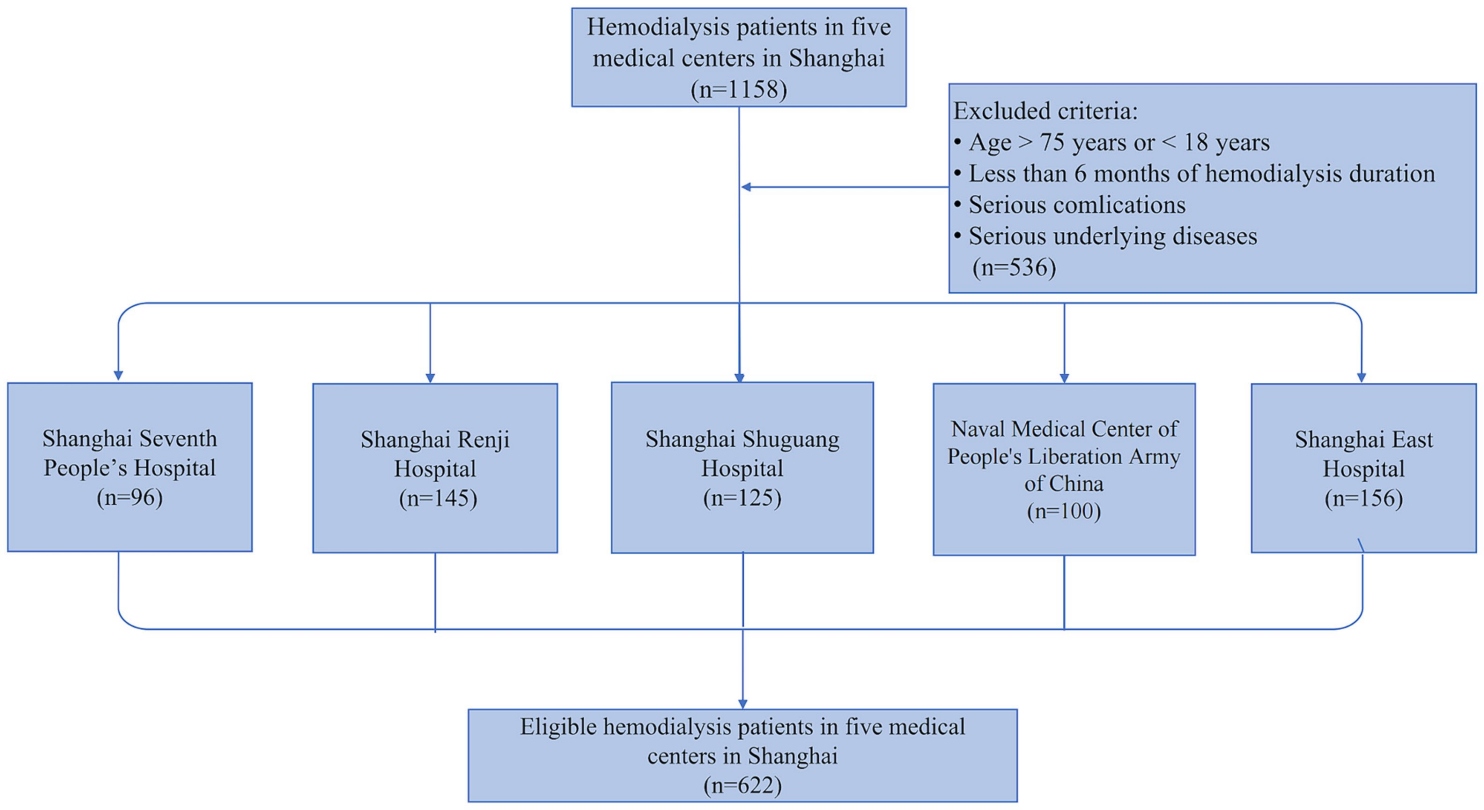
Flowchart of the external validation study. 1,158 adult hemodialysis patients from five medical centers in Shanghai were screened in our external validation study, and 622 patients were finally enrolled based on the exclusion criteria.

This cross-sectional study in MHD patients for external validation is conducted on five different medical centers in Shanghai, including Shanghai Seventh People’s Hospital, Shanghai Renji Hospital, Shanghai Shuguang Hospital, Naval Medical Center of People’s Liberation Army of China, and Shanghai East Hospital. We integrated the external validation cohort according to the following inclusion criteria: age range 18–75 years; maintenance hemodialysis for at least 6 months; contained all the metrics needed for the predictive model; and consented to participate in all aspects of the study. The exclusion criteria were as follows: pregnancy; thyroid dysfunction; corticosteroid or immunosuppressive medication; systemic infections, cardiovascular events, operations, trauma, and tumors for which a patient had received radiotherapy or chemotherapy within 3 months; active communicable diseases; patients enrolled in other clinical studies. Ultimately, 622 individuals were eligible for this current study. Of these, 96 participants were enrolled from Shanghai Seventh People’s Hospital, 145 participants were from Shanghai Renji Hospital, 125 participants were from Shanghai Shuguang Hospital, 100 participants were from Naval Medical Center of People’s Liberation Army of China, and 156 participants were from Shanghai East Hospital (Figure 1). It’s worth mentioning that these populations are temporally or spatially independent of the populations used in the previous predictive modeling exercise (22). This study was conducted according to the guidelines of the Helsinki Declaration and written informed consent was obtained from all patients. The study protocol was approved by the Human Research Ethics Committee of Shanghai East Hospital Affiliated with Tongji University School of Medicine (ChiCTR2000038127).

### 2.2 Demographic and laboratory measurements

Information and demographic data were retrieved by a trained interviewer using a standard questionnaire, including age, gender, education level, height, weight, primary renal disease, comorbidities (hypertension, diabetes, hyperlipidemia, stroke, and cardiovascular disease), systolic blood pressure, diastolic blood pressure, MAMC and DPI. MAMC was calculated by using the following formula: MAMC = arm circumference (mm) – 3.14 × triceps skin-fold thickness (mm) (29). DPI was estimated by using a 3-day dietary questionnaire to record the dietary intake of each patient for three consecutive days (including two working days and one weekend) (30). BMI was calculated by dividing the dry weight of dialysis patients by their height^2^.

Blood samples were collected after 12 h of fasting. Biochemistry data including serum albumin (g/L), TG (mmol/L), TC (mmol/L), vitamin D (ng/mL), NT-proBNP (ng/L), serum prealbumin (mg/L), serum bilirubin (mmol/L), alanine aminotransferase (U/L), aspartate aminotransferase (U/L), serum creatinine (mmol/L), blood urea nitrogen (mmol/L), serum uric acid (mmol/L), high-density lipoprotein cholesterol (HDL-c, mmol/L), low-density lipoprotein cholesterol (LDL-c, mmol/L), fasting blood glucose (mmol/L), plasma calcium (mmol/L), plasma magnesium (mmol/L), plasma phosphorous (mmol/L), serum iron (mmol/L), serum ferritin (ng/mL), intact parathyroid hormone (iPTH, pg/mL), lymphocyte count (10^9^/L), hemoglobin (g/L), and C-reactive protein (CRP, mg/L) were collected. Serum albumin, serum bilirubin, alanine aminotransferase, aspartate aminotransferase, serum creatinine, blood urea nitrogen, serum uric acid, TC, TG, HDL-c, LDL-c, fasting blood glucose, plasma calcium, plasma magnesium, plasma phosphorous, serum iron, serum ferritin, hemoglobin, CRP, and iPTH were measured by enzymatic colorimetry; serum prealbumin was measured by immunoturbidimetry; vitamin D was measured by competition method; and NT-proBNP was measured by double antibody sandwich method. All central laboratory data testing methods were harmonized.

### 2.3 Definition of PEW

The diagnostic criteria for the concept of PEW, which was introduced by the ISRNM in 2008, are as follows (At least three of the following four categories must fulfill the diagnostic requirements for PEW associated with kidney disease, and each criterion should be documented at least three times, preferably at 2–4 week intervals): (1) serum chemistry: serum albumin < 38 g/L, serum prealbumin < 0.3 g/L, or serum cholesterol < 1 g/L; (2) body mass: BMI < 23 kg/m^2^, unintentional 5% weight loss over 3 months or 10% weight loss over 6 months, and total body fat percentage < 10%; (3) muscle mass: reduced 5% muscle mass over 3 months or 10% over 6 months, reduction of MAMC area over 10% about 50th percentile of reference population, and creatinine appearance; (4) DPI: unintentional low DPI < 0.80 g/kg per day for at least 2 months for dialysis patients or DPI < 0.60 g/kg per day for patients with CKD stages 2–5 (14).

### 2.4 Prediction model

Chen et al. (22) established a novel clinical prediction model of PEW for adult hemodialysis patients to simplify the diagnosis, consisting of the following seven main factors, BMI, serum albumin, TC, gender, TG, vitamin D, and NT-proBNP.

### 2.5 Statistical analysis

The sample size was derived based on the available data. Descriptive statistics were reported as frequencies and proportions for categorical variables, and median (IQR) or mean (SD) for continuous variables. The scores of all patients in the validation set were calculated from the already established Nomogram (31). The external validation and performance of the prediction model were quantified by three aspects: discrimination, calibration, and decision curve analysis (DCA). All probabilities were two-tailed, and the level of significance was set at 0.05. Statistical analysis was performed using SPSS (version 23.0) and RStudio (version 2021.09.1 + 372).

#### 2.5.1 Discrimination

Area under the ROC Curve (AUC) and Concordance Index (C-index) are both metrics commonly used to evaluate the performance of binary classification models, measuring the predictive power and discrimination of the model (32). We calculated these two metrics separately for the prediction model using the R software, including 95% confidence interval (CI).

#### 2.5.2 Calibration

Calibration is the agreement between predicted probabilities and observed endpoints. The calibration curve can help us visualize whether the predicted probability of the model is consistent with the actual observations, and is still the preferred metric for evaluating the calibration of the model (32). Moreover, calibration can also be assessed by the Hosmer–Lemeshow test. If a *p*-value <0.05 is obtained, it means that there is a difference between the predicted and true values of the model. We applied both metrics to demonstrate that the predictive model is well-calibrated.

#### 2.5.3 Clinical impact curve

We plotted the Clinical Impact Curve and loss-to-benefit ratios to ascertain the threshold probability of delivering a higher net benefit.

## 3 Results

### 3.1 Descriptive characteristics of the external validation cohort

A total of 622 eligible patients were selected for inclusion. Of these, 287 (46.14%) participants were diagnosed with PEW according to the ISRNM diagnostic criteria (14). Table 1 summarizes the detailed characteristics of the 622 patients, including all predictors of the novel prediction model. Moreover, the primary diseases of all participants are described in Table 2.

**Table 1:** Characteristic description of external validation population.

	Validation cohort (*n* = 622)
Age (years)	62.00 (53.00, 68.00)
Male, *n* (%)	380 (61.09)
PEW, *n* (%)	287 (46.14)
Hospital, *n* (%)	
1	96 (15.43)
2	145 (23.31)
3	125 (20.09)
4	100 (16.08)
5	156 (25.08)
Education level, *n* (%)	
Junior school or below	307 (49.36)
High school	177 (28.46)
College or above	138 (22.19)
Smoking, *n* (%)	162 (26.05)
Drinking, *n* (%)	78 (12.54)
Hypertension, *n* (%)	487 (78.30)
Diabetes, *n* (%)	175 (28.14)
Hyperlipidemia, *n* (%)	94 (15.11)
CVD, *n* (%)	117 (18.81)
Stroke, *n* (%)	52 (8.36)
Dialysis duration time (months)	60.00 (24.00, 118.75)
SBP (mmHg)	138.96 ± 21.26
DBP (mmHg)	76.94 ± 11.77
BMI (kg/m^2^)	22.16 ± 3.81
MAMC (cm)	21.06 ± 2.55
DPI (g/kg/day)	0.88 ± 0.35
Serum albumin (g/L)	38.90 ± 4.31
Serum prealbumin (mg/L)	304.27 ± 82.59
TC (mmol/L)	3.76 (3.16, 4.48)
TG (mmol/L)	1.61 (1.13, 2.38)
Vitamin D (ng/mL)	15.08 (11.00, 20.99)
NT-proBNP (ng/L)	4,122.25 (1,872.00, 13,806.13)
Hemoglobin (g/L)	109.51 ± 16.90
Lymphocyte count (10^9^/L)	1.13 (0.87, 1.48)
CRP (mg/L)	1.83 (0.63, 5.46)
FBG (mmol/L)	6.23 (5.17, 8.12)
HbA1c (%)	5.60 (5.23, 6.40)
ALT (U/L)	11.29 ± 10.28
AST (U/L)	16.63 ± 6.57
BUN (mmol/L)	25.04 ± 13.75
Scr (μmol/L)	936.85 (735.25, 1,136.50)
SUA (μmol/L)	436.50 (361.78, 497.00)
eGFR (mL/min/1.73 m^2^)	4.00 (3.54, 5.00)
HDL-c (mmol/L)	0.95 (0.78, 1.17)
LDL-c (mmol/L)	2.03 (1.56, 2.65)
Plasma calcium (mmol/L)	2.31 (2.19, 2.46)
Plasma phosphorous (mmol/L)	1.79 (1.37, 2.25)
Serum iron (μmol/L)	12.10 (8.80, 19.30)
Plasma magnesium (mmol/L)	1.09 (0.99, 1.19)
Serum ferritin (ng/mL)	78.50 (30.18, 259.00)
iPTH (pg/mL)	202.00 (80.35, 405.75)

Datas are median (IQR) or mean ± SD unless otherwise stated. Mean ± SD was presented for variables with normal distribution while median (IQR) was presented for variables with abnormal distribution. 1, Shanghai Seventh People’s Hospital; 2, Shanghai Renji Hospital; 3, Shanghai Shuguang Hospital; 4, Naval Medical Center of People’s Liberation Army of China; 5, Shanghai East Hospital; ALT, alanine aminotransferase; AST, aspartate aminotransferase; BMI, body mass index; BUN, blood urea nitrogen; CRP, C-reactive protein; CVD, cardiovascular disease; DBP, diastolic blood pressure; DPI, dietary protein intake; FBG, fasting blood glucose; HDL-c, high-density lipoprotein cholesterol; LDL-c, low-density lipoprotein cholesterol; MAMC, mid-arm muscle circumference; PEW, Protein Energy Wasting; NT-proBNP, N-terminal pro-B-Type natriuretic peptide; iPTH, intact parathyroid hormone; SBP, systolic blood pressure; Scr, serum creatinine; SUA, serum uric acid; TC, total cholesterol; TG, triglycerides.

**Table 2:** Primary diseases of hemodialysis patients.

Primary diseases	*n* (%)
**Primary kidney diseases**	
Chronic glomerulonephritis	154 (24.76)
**Secondary kidney diseases**	
Hypertensive nephropathy	131 (21.06)
Diabetic nephropathy	118 (18.97)
Obstructive nephropathy	21 (3.38)
Rheumatic immune system diseases	16 (2.57)
Hyperuricemia nephropathy	14 (2.25)
Chronic pyelonephritis	6 (0.96)
Drug-induced nephropathy	6 (0.96)
**Congenital kidney diseases**	
Polycystic kidney disease	33 (5.31)
**Etiology unknown**	123 (19.77)

### 3.2 Performance of the model in the external validation

#### 3.2.1 Discrimination

**Figure 2:**
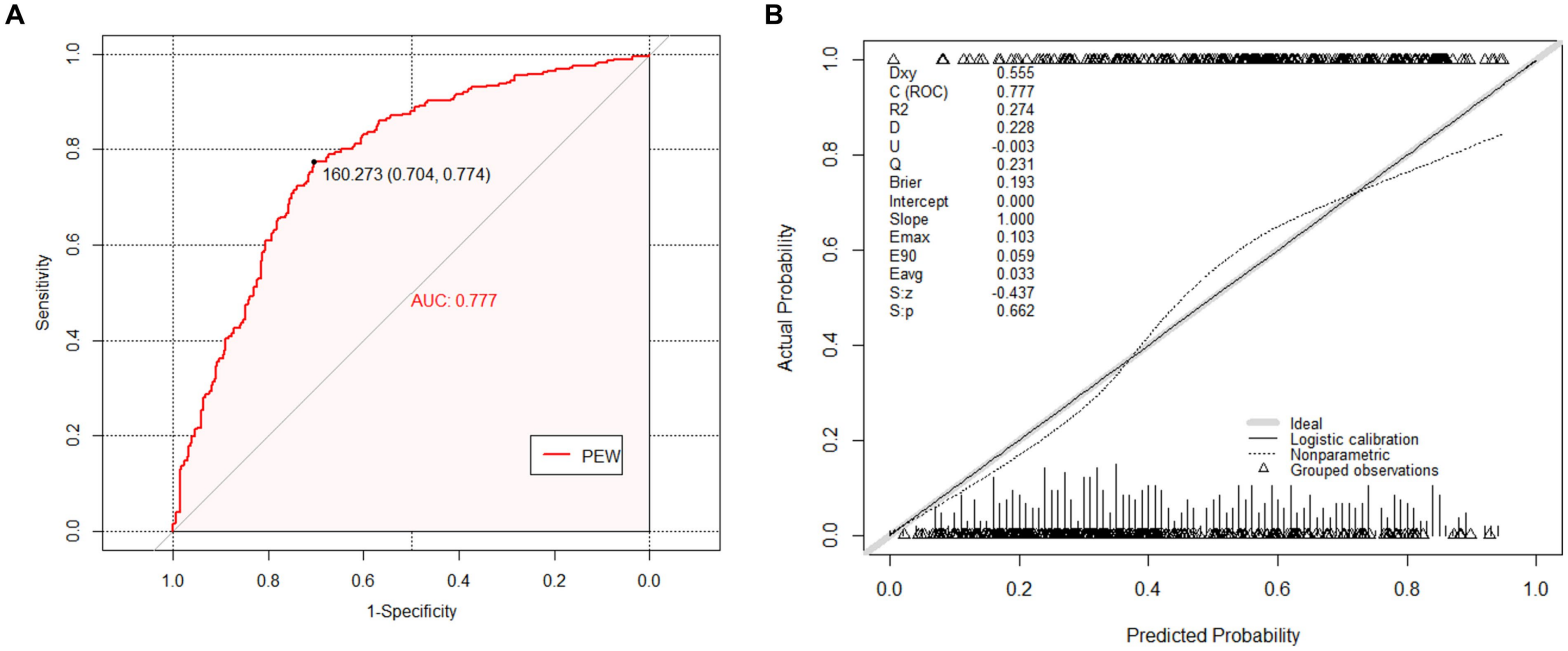
The receiver operating characteristic (ROC) curves and calibration curves of the external validation cohort. **(A)** The ROC curves of nomogram in external validation cohort (AUC = 0.777, 95% CI 0.741–0.814). The nomogram was the prediction model for predicting risk of Protein Energy Wasting (PEW) in maintenance hemodialysis (MHD) patients that we want to verify. **(B)** The calibration curves of the nomogram in external validation cohort. It demonstrated the clinical effectiveness of the nomogram by comparing the predicted risk and actual risk assessment. The calibration curves showed good agreement between prediction and observation in the probability of PEW.

In our cohort, the AUC, which assesses discrimination power, was 0.777 (95% CI: 0.741–0.841) (shown in Figure 2A). In addition, the C-index for evaluating the accuracy of the predictions of the model was 0.777. Both results demonstrated that the prediction model is well-discriminated.

#### 3.2.2 Calibration

Regarding calibration, the diagnostic nomogram yielded a calibration curve, which indicated high consistency between prediction and observation in the probability of PEW (Figure 2B). According to the calibration plot, the Brier score was 0.193, reflecting good accuracy and robustness. Moreover, U refers to the unreliability test, which assumes that there is no correlation between the predicted value and the true value, and the calibration is better when the value is closer to 0. The corresponding *p*-value was the following: S: *p* refers to the *p*-value of the Spiegelhalter Z-test, when S: *p* > 0.05 indicates that through the calibration test. The prediction model had a strong calibration ability with an S: *p* of 0.662, indicating diagnostic accuracy. Meanwhile, the average difference between projected and actual values (*E*_avg_) and maximal absolute differences between predicted and actual values (*E*_max_) in the predicted and calibrated probabilities were given in the plot. The Hosmer–Lemeshow test (*p* = 0.108) revealed that the model had favorable coherent properties.

#### 3.2.3 Clinical impact curve

**Figure 3:**
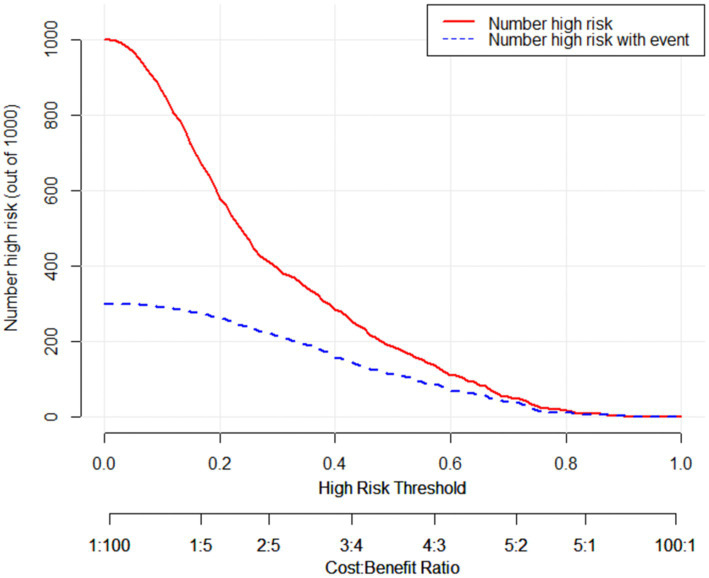
The clinical impact curve of the external validation cohort. The horizontal axis represents the probability threshold, while the vertical axis represents the number of people. The red line represents the number of people considered high-risk by the model at different probability thresholds. The blue line represents the number of people identified as high-risk by the model and who truly have Protein Energy Wasting (PEW) at different probability values. At the bottom, there is a loss-to-benefit ratio, which indicates the proportion of loss to benefit at different probability thresholds. These findings demonstrate that a threshold probability of 0.6 yields a beneficial outcome.

To further illustrate that the model had a favorable clinical benefit, we also plotted the Clinical Impact Curve (Figure 3), which included the loss-to-benefit ratios at each threshold probability. We found that the highest benefit was obtained at a threshold probability of 60%, which was also consistent with the threshold probability taken for model development (22).

#### 3.2.4 Comparison of two diagnostic methods

According to Table 3, the *p* value of the McNemar test was 0.536 (*p* > 0.05). The results showed that there was no significant difference between the gold standard diagnostic method and the novel model.

**Table 3:** Comparison between gold standard diagnostic method and prediction model.

		Prediction model	
		Negative	Positive
Gold standard	Negative	247	88
	Positive	79	208
*χ*^2^ *=* 0.383			
*p =* 0.536			

McNemar test was used to compare the gold standard diagnostic method and prediction model. The results showed that there was no significant difference between the two diagnostic methods (*p* < 0.05).

#### 3.2.5 The diagnostic characteristic of the prediction model

The diagnostic features of the model are listed in Table 4. The ≥50% probability threshold defined approximately 57% of the population as high-risk, with a sensitivity of 80.14% (95% CI: 75.05–84.60%), specificity of 62.69% (95% CI: 57.26–67.88%), PPV of 64.79% (95% CI: 61.29–68.14%), and NPV of 78.65% (95% CI: 74.22–82.5%). The maximum Youden index indicated a threshold of ≥60%. Considering this cut point, about 48% of the total population was categorized as high-risk individuals with a sensitivity of 72.47% (95% CI: 66.92–77.56%), specificity of 73.73% (95% CI: 68.67–78.37%), PPV of 70.27% (95% CI: 66.09–74.14%), NPV of 75.77% (95% CI: 71.94–79.22%), positive LR of 2.76 (95% CI: 2.28–3.35) and negative LR of 0.37 (95% CI: 0.31–0.46).

**Table 4:** The clinical performance of the updated model for PEW.

Probability threshold (%)	≥50	≥60	≥70	≥80
Validation cohort (*N* = 622, PEW = 287)				
High-risk population, %	57.07	47.59	41.00	31.67
PEW, %	64.79	70.27	71.37	71.57
Sensitivity, % (95% CI)	80.14 (75.05–84.60)	72.47 (66.92–77.56)	63.41 (57.55–69.00)	49.13 (43.21–55.07)
Specificity, % (95% CI)	62.69 (57.26–67.88)	73.73 (68.67–78.37)	78.21 (73.40–82.51)	83.28 (78.85–87.12)
PPV, % (95% CI)	64.79 (61.29–68.14)	70.27 (66.09–74.14)	71.37 (66.65–75.67)	71.57 (65.86–76.67)
NPV, % (95% CI)	78.65 (74.22–82.50)	75.77 (71.94–79.22)	71.39 (67.96–74.59)	65.65 (62.81–68.37)
LR positive (95% CI)	2.15 (1.85–2.5)	2.76 (2.28–3.35)	2.91 (2.33–3.63)	2.94 (2.25–3.84)
LR negative (95% CI)	0.32 (0.25–0.41)	0.37 (0.31–0.46)	0.47 (0.40–0.55)	0.61 (0.54–0.69)
Youden index, % (95% CI)	42.83 (32.31–52.48)	46.2 (35.59–55.93)	41.62 (30.95–51.51)	32.41 (22.06–42.19)
AUC (95% CI)	0.714 (0.677–0.749)	0.731 (0.694–0.766)	0.708 (0.671–0.744)	0.662 (0.623–0.699)

All analysis was done based on survey data analysis. PEW, Protein Energy Wasting; PPV, positive predictive value; NPV, negative predictive value; LR, likelihood ratio; AUC, Area Under Curve.

## 4 Discussion

In the current study, a total of 622 samples from five different medical centers were collected to externally validate the predictive model for PEW. As seen in our results, the predictive model provided excellent discrimination and calibration, and was able to identify whether a hemodialysis patient was PEW at an earlier stage. We also indicated that the 60% threshold probability had a high capacity to predict the risk of PEW with a high clinical benefit. In our external validation set, the incidence of PEW was 46.14%, which was almost the same as the incidence of PEW in the model development set. Although the AUC was 0.777, which was slightly smaller than the internal validation AUC of 0.85, this result was expected during external validation (22, 23, 33). The possible reasons for this discrepancy are analyzed as follows: first, the sample size of the development cohort of the model is comparatively small; second, the data distribution of the development cohort and the validation cohort is very similar during internal validation, which may lead to overfitting, while the distribution of the population characteristics of the external validation cohort differs from that of the development cohort.

Accompanied with the development of science and technology, predictive models are increasingly used in kidney diseases. The quality and clinical influence of these prediction models have fallen short of their intended potential. One reason for this is that despite the development of many models, only part of them have been externally validated (34), and the field of nephrology is no exception (21). Given the number of forecasting models developed, the proportion of studies that address external validation is small. A quick PubMed search revealed 84,032 studies on predictive modeling, of with only 4,309 (5%) referring to external validation in the title or abstract (21). External validation is needed to assess the repeatability and generalizability of the model (35, 36). Regarding the method of external validation, the score of all patients in the validation cohort is calculated from the predictive model that has been built. It is worth noting that the weights for the indicators are based on the already established model, rather than re-running a multifactorial analysis of the validation cohort, which would otherwise only prove that these variables do remain predictors in the external validation cohort (31).

As no single diagnostic marker or tool is most effective in determining whether a patient was PEW, clinical studies focusing on PEW will necessarily need to incorporate one or more nutrition-related surrogates for the diagnosis. The diagnostic criteria proposed by the 2008 ISRNM are four components: low biochemical markers (serum albumin, prealbumin, or TC); generalized adiposity or weight loss; loss of muscle mass; and inadequate protein or energy intake ratios (14). In 2014, Moreau-Gaudry et al. introduced a new simplified assessment method that used serum creatinine corrected for body surface area (sCr/BSA) as a surrogate for muscle loss over time (37, 38). The new marker has the advantage of being easy to measure and enabling earlier diagnosis of protein depletion, rather than having to wait 3–6 months for muscle mass loss to be detected (37). The study showed that the new PEW-score 2014, which incorporated sCr/BSA, identified a higher proportion of dialysis PEW patients than the PEW-ISRNM 2008. Although the PEW-score 2014 was more clinically relevant as it provided more timely information, its correlation with premature mortality would need to be proven in larger studies, which were not yet available to further prove it, and this score only predicted all-cause mortality in European patients undergoing hemodialysis, with ethnicity, habitus, and social background contributing to significant variations in the nutritional status and parameters of patient populations across countries (37).

Additionally, in 2021, the Geriatric Nutritional Risk Index (GNRI), launched initially as a modified nutritional exposure index for older adults, drew attention in assessing PEW (39). Compared to the Subjective Global Assessment (SGA) and the Malnutrition Inflammation Score (MIS), which require subjective assessment, the GNRI is a brief, objective nutritional measure that only involves two constituents (serum albumin concentration and actual-to-ideal body weight ratio), and it has already as validated as an effective assessment tool for ESRD patients in Asia (39). Beberashvili et al. conducted a comparison of MIS and GNRI for hemodialysis patients and observed that there was less agreement between observers for MIS than for GNRI (40). However, variations in daily energy and protein intake were correlated with the MIS rather than the GNRI. In terms of this study, moreover, it suggests that only MIS is an important risk factor for death, and thus MIS is likely to be a more comprehensive tool than the GNRI (40).

For the diagnosis of PEW, our previous study proposed a novel model for predicting the risk of PEW in adult hemodialysis patients, and its validity was confirmed by internal validation (22). The study included 380 adult hemodialysis patients who had been on continuous dialysis for more than 6 months in the hemodialysis centers of several tertiary hospitals in Shanghai, and incorporated seven indicators as predictors: albumin, TC, TG, BMI, gender, vitamin D, and NT-proBNP (22). In contrast to the diagnostic criteria proposed in 2008, the model added four new independent influences: gender, TG, vitamin D, and NT-proBNP. The study showed that female patients had a higher risk of developing PEW than male patients, which might be related to sex hormones and different adipokines distribution (22). In addition, TG was found to be protective in the development of PEW. Notably, it had been implicated that plasma TG n-3 polyunsaturated fatty acids (PUFAs) were linked to both an inferior level of inflammatory markers and improved nutritional condition in patients with MHD. Furthermore, TG n-6 PUFAs have shown a positive correlation with higher serum albumin levels and increased grip strength (41). For vitamin D, it performs an influential part in the regulation of skeletal muscle metabolism. Low levels of vitamin D notably increase the mortality in MHD patients with PEW (42). Besides, NT-proBNP is a possible independent biomarker for the occurrence of PEW in patients with MHD, probably because it is negatively correlated with the amount of body fat and dramatically increases the incidence of PEW in adult hemodialysis patients (43). Metrics such as discrimination and calibration revealed that the model has good predictive ability and clinical utility. It was more accessible and objective, facilitating early identification and intervention of PEW in MHD patients by clinical physicians. Unfortunately, it was not externally validated to demonstrate a high clinical translation rate.

For the strengths of our study, the main points are as follows: Firstly, our study population is independent of the model development set, which is more heterogeneous and has a larger sample size, making the results more convincing. Secondly, the multicenter design makes the data more extensive, which allows a better assessment of the generalizability of the model. Lastly, we use a combination of temporal and spatial validation, which is more comprehensive and prospective.

Nevertheless, our study also contains limitations. At first, this is a cross-sectional study, which is affected by external factors to some extent, and prospective studies are required to provide more instructive information. Furthermore, previous studies have developed predictive models for PEW in peritoneal dialysis patients (26), and our study could be compared and optimized to allow for a larger population-based prediction of PEW. Finally, the population in our study was from Shanghai. Although bone mineral metabolism, cardiac function are now routinely assessed for complications in dialysis patients and have become hemodialysis standard operating procedure (SOP) in China, it’s important to note that many traditional hemodialysis clinics globally may not routinely measure biomarkers such as vitamin D and NT-proBNP. To address this, we may need to expand the sample size and consider a broader geographic region for further improvements.

## 5 Conclusion

This external validation study demonstrated the feasibility of the novel PEW risk prediction model which was previously developed and established by our center. Its diagnostic validity was in high agreement with the PEW diagnostic gold standard proposed by ISRNM in 2008. It has the potential to replace the current gold standard for discriminating PEW in adult hemodialysis patients. It simplifies the gold standard, aiding in early identification and prevention of PEW, and improves long-term prognosis and survival.

## Data Availability

The original contributions presented in the study are included in the article/supplementary materials, further inquiries can be directed to the corresponding authors.
